# Plasma Levels of Middle Molecules to Estimate Residual Kidney Function in Haemodialysis without Urine Collection

**DOI:** 10.1371/journal.pone.0143813

**Published:** 2015-12-02

**Authors:** Enric Vilar, Capella Boltiador, Jonathan Wong, Adie Viljoen, Ashwini Machado, Arani Uthayakumar, Ken Farrington

**Affiliations:** 1 Renal Unit, Lister Hospital, Hertfordshire, Stevenage, United Kingdom; 2 Department of Postgraduate Medicine, University of Hertfordshire, United Kingdom; 3 Department of Pathology, Lister Hospital, Stevenage, United Kingdom; 4 University of the West of England, Bristol, United Kingdom; Medical University of Graz, AUSTRIA

## Abstract

**Background:**

Residual Kidney Function (RKF) is associated with survival benefits in haemodialysis (HD) but is difficult to measure without urine collection. Middle molecules such as Cystatin C and β2-microglobulin accumulate in renal disease and plasma levels have been used to estimate kidney function early in this condition. We investigated their use to estimate RKF in patients on HD.

**Design:**

Cystatin C, β2-microglobulin, urea and creatinine levels were studied in patients on incremental high-flux HD or hemodiafiltration(HDF). Over sequential HD sessions, blood was sampled pre- and post-session 1 and pre-session 2, for estimation of these parameters. Urine was collected during the whole interdialytic interval, for estimation of residual GFR (GFR_Residual_ = mean of urea and creatinine clearance). The relationships of plasma Cystatin C and β2-microglobulin levels to GFR_Residual_ and urea clearance were determined.

**Results:**

Of the 341 patients studied, 64% had urine output>100ml/day, 32.6% were on high-flux HD and 67.4% on HDF. Parameters most closely correlated with GFR_Residual_ were 1/β2-micoglobulin (r^2^ 0.67) and 1/Cystatin C (r^2^ 0.50). Both these relationships were weaker at low GFR_Residual_. The best regression model for GFR_Residual_, explaining 67% of the variation, was:

$${GFR}_{Residual}=160.3\;\cdot \left(\frac{1}{\beta 2m}\right)-4.2$$

Where *β2m* is the pre-dialysis β2 microglobulin concentration (mg/L). This model was validated in a separate cohort of 50 patients using Bland-Altman analysis. Areas under the curve in Receiver Operating Characteristic analysis aimed at identifying subjects with urea clearance≥2ml/min/1.73m^2^ was 0.91 for β2-microglobulin and 0.86 for Cystatin C. A plasma β2-microglobulin cut-off of ≤19.2mg/L allowed identification of patients with urea clearance ≥2ml/min/1.73m^2^ with 90% specificity and 65% sensitivity.

**Conclusion:**

Plasma pre-dialysis β2-microglobulin levels can provide estimates of RKF which may have clinical utility and appear superior to cystatin C. Use of cut-off levels to identify patients with RKF may provide a simple way to individualise dialysis dose based on RKF.

## Introduction

There is increasing evidence that residual kidney function (RKF) is major contributor to long-term survival in both HD[1, 2] and peritoneal dialysis[3, 4]. Dialysis strategies that protect RKF may therefore be beneficial. However measuring RKF in HD has not been practiced widely because of the inconvenience of having to perform urine collections to allow clearance to be estimated.

It is known that RKF is the major determinant of the plasma levels of many middle molecules even in the dialysis population. On this basis it has been proposed that plasma levels of middle molecules might be useful as markers of RKF, avoiding the need for urine collection[5, 6]. Cystatin C, β2-microglobulin, and beta trace protein have all been suggested as potential candidate molecules [5–9].

Cystatin C is a lysosomal protease and cysteine proteinase inhibitor produced by all nucleated cells [10, 11]. At 13.3 kDa, it is of similar size to β2 microglobulin (12.6kDa). Being freely filtered by glomeruli and metabolised by proximal tubules[12], Cystatin C may be used as an alternative to creatinine as a marker of glomerular filtration rate (GFR) without the need to correct for muscle bulk, sex and race both in subjects with normal and moderately impaired kidney function[13–15]. However, although Hoek et al have reported plasma Cystatin C levels to be related to RKF in HD patients[6], its value as a plasma predictor of GFR remains unclear in this setting. Similarly, a close relationship has been found between β2-microglobulin plasma concentration and RKF in HD [16] and peritoneal dialysis [17, 18]. This relationship also holds in the context of haemodiafiltration (HDF), a technique that enhances middle molecule clearance and has now been shown to be improve survival in both retrospective [19, 20] and prospective randomised control trials in HD[21, 22]. HDF involves the use of high convective volumes to maximise middle molecule clearance, Even in this setting however, RKF is the major determinant of plasma levels of β2-microglobulin. Β2-microglobulin is therefore a potential plasma marker of RKF and at 11.6kDa is of similar size to Cystatin C. Kinetic studies of generation rate and non-renal clearance suggest that there is lower inter-individual variation for β2-microglobulin compared to Cystatin C and this suggests a potential closer relationship between plasma β2-microglobulin levels and RKF than for Cystatin C[23, 24].

In this study we aimed to determine the utility of plasma β2-microglobulin and Cystatin C concentrations for estimation of RKF in patients on HD. Using regression equations, we aimed to determine predictive equations for RKF in patients on haemodialysis based on plasma levels of β2-microglobulin and Cystatin C. We also aimed to determine whether a cut-off middle-molecule level may be used to identify patients who have significant RKF which might allow modification of dialysis doses for incremental dialysis[25].

## Methods

### Study design and setting

The study was performed with approval from the Hertfordshire (UK) Research Ethics Committee (Reference 09/H0311/07) and in accordance with the Declaration of Helsinki. The ethics committee reviewed the consent process and agreed that verbal but not written consent would be required from subjects as no additional blood draws would be required due to sampling of blood being simultaneous with routine monthly samples and three other markers of renal function already being tested routinely (urea, creatinine, β2-microglobulin) with the only additional marker being cystatin C. For inclusion patients were required to be >18 years and on regular dialysis at the East and North Herts NHS Trust. There were no exclusion criteria. Patients were provided with an information sheet >24hours prior to study participation and their verbal consent was documented in both hospital medical notes and study records.

341 patients established on chronic thrice weekly high-flux HD or on-line HDF were recruited as a test cohort. All subjects had a measure of RKF and sampling of plasma pre and post-dialysis urea, creatinine, cystatin C and β2 microglobulin to determine predictive models for estimating RKF from plasma levels. Refusal to participate was not formally recorded but was very low (<10%) as the study was performed simultaneously with routine monthly dialysis blood samples and urine collections for RKF were routinely performed in the dialysis unit in all uric patients.

Predictive models from this test cohort were validated in an entirely separate cohort (n = 50) of patients from the same centre. This sample was recruited at a different time point while ensuring zero cross-over between patients in the two groups.

### Haemodialysis and on-line haemodiafiltration procedures

All patients were dialysed using Fresenius 5000 series or Nikisso HD machines using high-flux HD or online HDF. Where HDF was used it was used as a permanent treatment modality and switching between high-flux HD and HDF on a session-by-session basis (eg. once a week HDF) was nor performed. Data recorded included blood flow (Q_B_), dialysis fluid flow (Q_D_), ultrafiltration volume(UF) and body size parameters on the first dialysis session of the week. Body Surface Area (Dubois formula) was calculated from the weight and height. Bicarbonate was used as a buffer, and ultrapure water used both for dialysis and replacement fluid for online HDF. 94% of patients used Fresenius FX-class high-flux dialysers. All dialysed using an incremental algorithm whereby target total Kt/V (Kt/V_Tota_l) at 1.2 was the sum of dialysis two-pool eKt/V (Kt/V_Dialysis_) and the equivalent Kt/V provided by RKF (Kt/V_Renal_) [1, 26, 27]. For online HDF, the HDF fraction was 0.35 of plasma flow rate and target HDF volume/session was 40% of Watson volume.

### Measurement of residual kidney function

Residual GFR (GFR_Residual_) was measured using the mean of urea and creatinine clearance calculated from interdialytic urine collection and then corrected to 1.73m^2^. Patients with <100ml/day urine output were regarded as having zero GFR_Residual_. Urea and creatinine clearance were calculated using the formula:

$$Clearance\left(ml/min\right)=\frac{2\cdot \left(U_{ID}\cdot V_{ID}\right)}{t_{ID}\cdot \left(C_{post_{HD1}}+C_{pre_{HD2}}\right)}$$

Where U_ID_ is the urinary concentration, V_ID_ is the urine collection volume (ml), t_ID_ is the collection duration (min), and C_post_HD1_ and C_pre_HD2_ are the plasma concentrations at the beginning and end of the urine collection.

### Sampling of blood for clearance markers

Over consecutive dialysis sessions (HD1 and HD2), blood samples were drawn pre- and post-HD1 and pre-HD2. HD1 was the dialysis session immediately following the weekend. Pre dialysis samples were drawn from the arterial needle immediately before dialysis. Post-dialysis samples were taken at the end of treatment with the blood pump slowed to 50ml/min for 30 seconds prior to sampling to reduce access recirculation. Samples were analysed for urea, creatinine, β2-microglobulin and Cystatin C. β2-microglobulin was measured using an Olympus AU640 (Beckman-Coulter) by immune-turbidimetric analysis. Cystatin C was measured on an IMMAGE^®^800 (Beckman-Coulter) analyser using DakoCytomation Cystatin C reagent (Dako Ltd, Cambridgeshire, UK) based on the non-competitive rate particle-enhanced nephelometric technology. Repeat analysis of patient samples showed< 3% inter-assay variation for both Cystatin C and β2-microglobulin.

### Dialysis Reduction Ratios

For each molecule, the reduction ratio during dialysis was calculated for HD1 as:

$${\left[\left({\text{C}}_{\text{pre}\_\text{HD}1}-{\text{C}}_{\text{post}\_\text{HD}1}\right)/{\text{C}}_{\text{pre}\_\text{HD}1}\;\right]}^{*}100\%.$$

Dialysis single pool Kt/V_urea_ was calculated using the Daugirdas 2^nd^ generation formula[28] and the equilibrated Kt/V_urea_ using the Tattersall transformation[29].

### Statistical methods

Demographic parameters in the test and validation cohorts were compared using T-tests, Mann-Whitney U tests and Fisher’s Exact test as appropriate.

Pre-HD levels of β2-microglobulin and Cystatin C, their reciprocals and their inter-dialytic rise were related to estimates of RKF (GFR_Residual_, urea and creatinine clearance) using Pearson’s correlation.

Predictive equations for RKF were determined based on pre-HD plasma concentrations of the above molecules using linear regression including (Eq 1, Results).

Receiver Operating Characteristic (ROC) analysis was used to determine cut-off plasma urea, creatinine, Cystatin C and β2-microglobulin concentrations which identified patients having varying degrees of RKF. Various levels of RKF were studied using urea clearance and GFR_Residual_ between 1 and 10ml/min/1.73m^2^ BSA. For each RKF cut-off level the plasma concentrations of Cystatin C and β2-microglobulin were determined that would yield a false positive rate of <10% (specificity 90%).Similar ROC analyses were performed to identify patients with GFR_Residual_ ≥ various levels using predicted GFR from the optimum regression equation (Eq 2, Results) as the test variable.

To determine the relationships of plasma Cystatin C and β2-microglobulin levels with overall urea-based adequacy measures, the correlations of Kt/V_Total_ with pre-dialysis plasma levels, and their reciprocals, were assessed.

### Validation of methods to predict RKF in a separate cohort

Demographics of the study cohort were compared to the validation cohort using parametric/non parametric tests as appropriate. The best derived predictive equation for GFR_Residual_ based on a single plasma middle molecule concentration was applied to the validation cohort (n = 50) and its performance assessed using the Bland-Altman technique. Additionally, the cut-off plasma concentration level of middle molecules that had been determined to identify patients with significant RKF were applied to the validation cohort to determine the sensitivity and specificity of the tests.

## Results

### Study population

341 subjects were recruited, of whom 111 were treated with high-flux HD and 230 using online HDF (Table 1). 36% of patients had RKF classified as zero. Demographic, clinical and dialysis characteristics in the test cohort are shown in **Table 1**. 35.1% of patients had residual kidney function of zero. Mean total Kt/V (eKt/V_urea_+ Kt/V_Renal_) was 1.33 ± SD 0.22 and mean dialysis eKt/V_urea_ was 1.14 ±SD 0.26 which reflects the incremental HD algorithm.

**Table 1:** Baseline characteristics and dialysis parameters of the study population.

	Descriptive	Mean, Median †, or percentage	Standard deviation, IQR*
Demographics	Age	62.7	17
	Males/Females (%)	65.7% M, 34.4% F	
	Weight (kg)	75.7	18.8
	Watson Volume (L)	38.7	8.1
	Body surface area (m^2^)	1.85	0.27
Dialysis parameters	Dialysis time (min)	190	32
	Blood flow [Qb] (ml/min)	329	65
	Dialysis fluid flow [Qd] (ml/min)	690	146
	High-flux HD/HDF (%)	32.6% High-flux HD, 67.4% HDF	
	Ultrafiltration (L)	1.66	1.02
	GFR (ml/min/1.73m^2^ BSA)	1.13†	4.44*
	Dialysis single pool Kt/V_urea_	1.35	0.29
	Dialysis eKt/V_urea_	1.14	0.26
	Residual renal equivalent Kt/V	0.10†	0.30*
	Total Kt/V_urea_ (Renal+Dialyser)	1.33	0.22

Total Kt/V_urea_ represents the sum of residual renal and dialyser clearance which was used for incremental dialysis calculations and as target Kt/V (see methods).

† Median.

* Interquartile range.

In the validation cohort, demographic parameters did not differ significantly from the test cohort and are reported in **Table 2**. 34% of the validation cohort had RKF classified as zero. Mean plasma concentrations are shown in Table 3. Pre-HD1 concentrations (Monday or Tuesday) were significantly higher than pre-HD2 (Wednesday or Thursday) with p<0.001 for each pair molecule (paired t tests).

**Table 2:** Demographics of the validation cohort.

	Descriptive	Mean, Median †, or percentage	Standard deviation, IQR*	Comparison to test cohort (p)
Demographics	Age	59.6	17.2	0.22
	Males/Females (%)	30% M, 70% F		0.63
	Weight (kg)	79.7	20.4	0.43
	Watson Volume (L)	40.2	8.6	0.21
	Body surface area (m^2^)	1.90	0.26	0.24
Dialysis parameters	Dialysis time (min)	210	29	0.03
	Blood flow [Qb] (ml/min)	329	65	0.53
	High-flux HD/HDF (%)	18% High-flux HD, 82% HDF		0.05
	Ultrafiltration (L)	1.7	1.00	0.99
	GFR (ml/min/1.73m^2^ BSA)	1.94†	4.38*	0.81
	Dialysis eKt/V_urea_	1.10	0.28	0.08
	Residual renal equivalent Kt/V	0.23†	0.43*	0.14
	Total Kt/V_urea_ (Renal+Dialyser)	1.35	0.30	0.76

† Median

* Interquartile range (IQR)

**Table 3:** Plasma levels of molecules at each study time-point

	Plasma levels (mean±SD)			Paired T test comparison of Pre-HD1 v Pre-HD2 (p)	Reduction ratio over HD1
	Pre-HD1	Post-HD1	Pre-HD2		
Urea (mmol/L)	22.7 ± SD6	7.1 ± SD2.7	19.2 ± SD5.1	<0.001	69%± SD7%
Creatinine (μmol/L)	827 ± SD256	296 ± SD103	734 ± SD233	<0.001	63%± SD8%
β2-microglobulin(mg/L)	26.6 ± SD8.4	9.2 ± SD3.4	24.6 ± SD7.4	<0.001	64%± SD10%
Cystatin C(mg/L)	5.3 ± SD1.1	2.6 ± SD0.6	5.2 ± SD1	<0.001	51%± SD11%

SD Standard Deviation

### Dialysis clearance of Cystatin C and β2-microglobulin

Reduction ratios for all molecules are shown in Table 2. Cystatin C reduction ratio correlated highly with β2-microglobulin reduction ratio (r^2^ = 0.70,p<0.001). In linear regression analysis controlling for eKt/V_urea_, treatment modality (high-flux HD v HDF) was an independent predictor of Cystatin C reduction ratio, HDF being associated with a 7% increase compared to high-flux HD (p < 0.001). Similarly HDF was associated with an 8% increase in β2-microglobulin reduction ratio (p<0.001).

Pre-dialysis concentration of β2 microglobulin was similar between the high-flux HD and HDF groups overall (mean 27.1±SD9.6mg/L v 26.3±SD7.8, p = 0.4) but in those with low level RKF (GFR<1ml/min/1.73m^2^ BSA) it was significantly lower (35.5±SDmg/L v 31.1±SD5.9, p<0.001).

### Relationship of residual kidney function to plasma Cystatin C and β2 microglobulin levels

**Fig 1:**
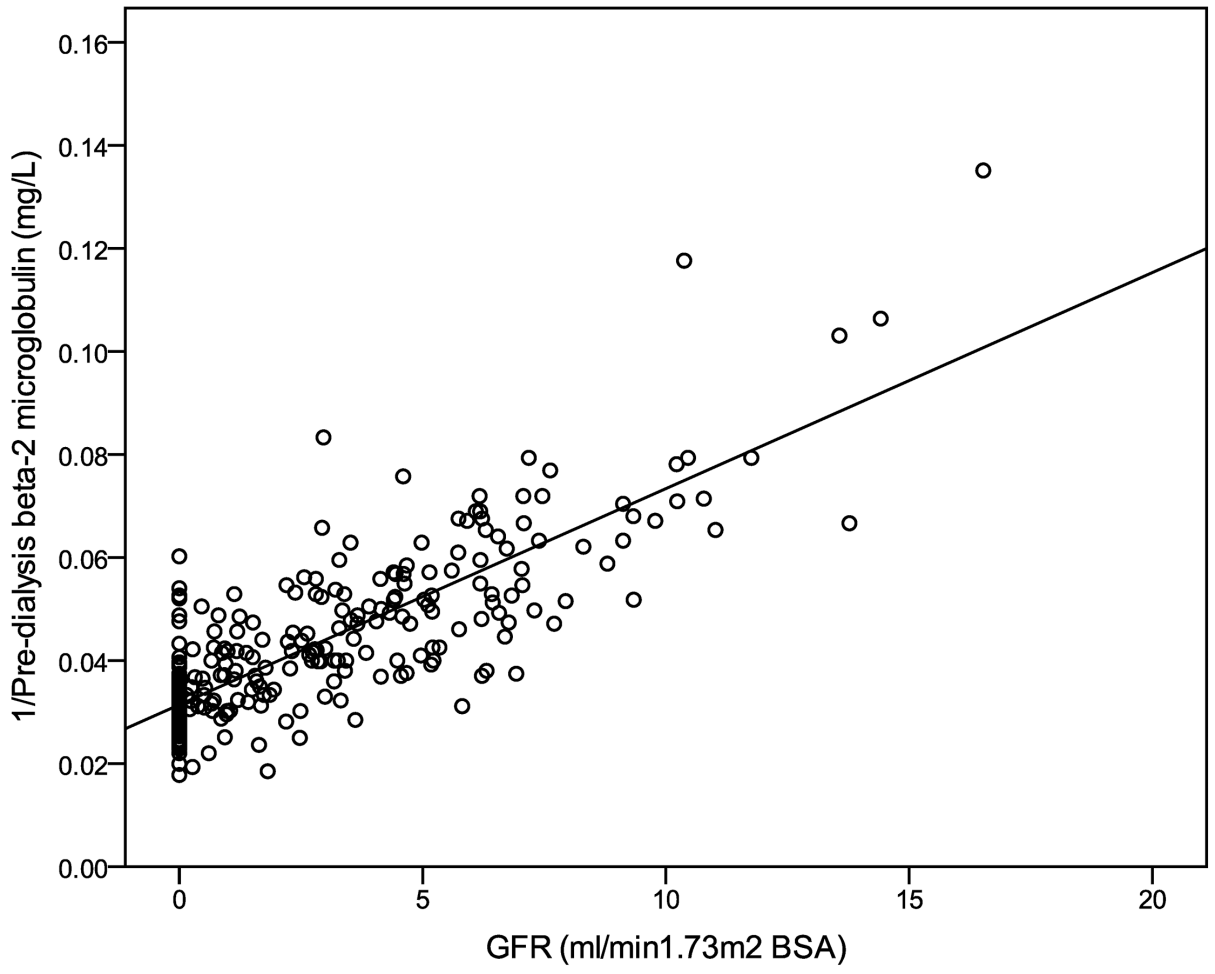
Relationship of GFR with the reciprocal of pre-dialysis β2 microglobulin levels. GFR shown is calculated from mean of urea and creatinine clearance. For the linear regression shown r2 was 0.67.

**Fig 2:**
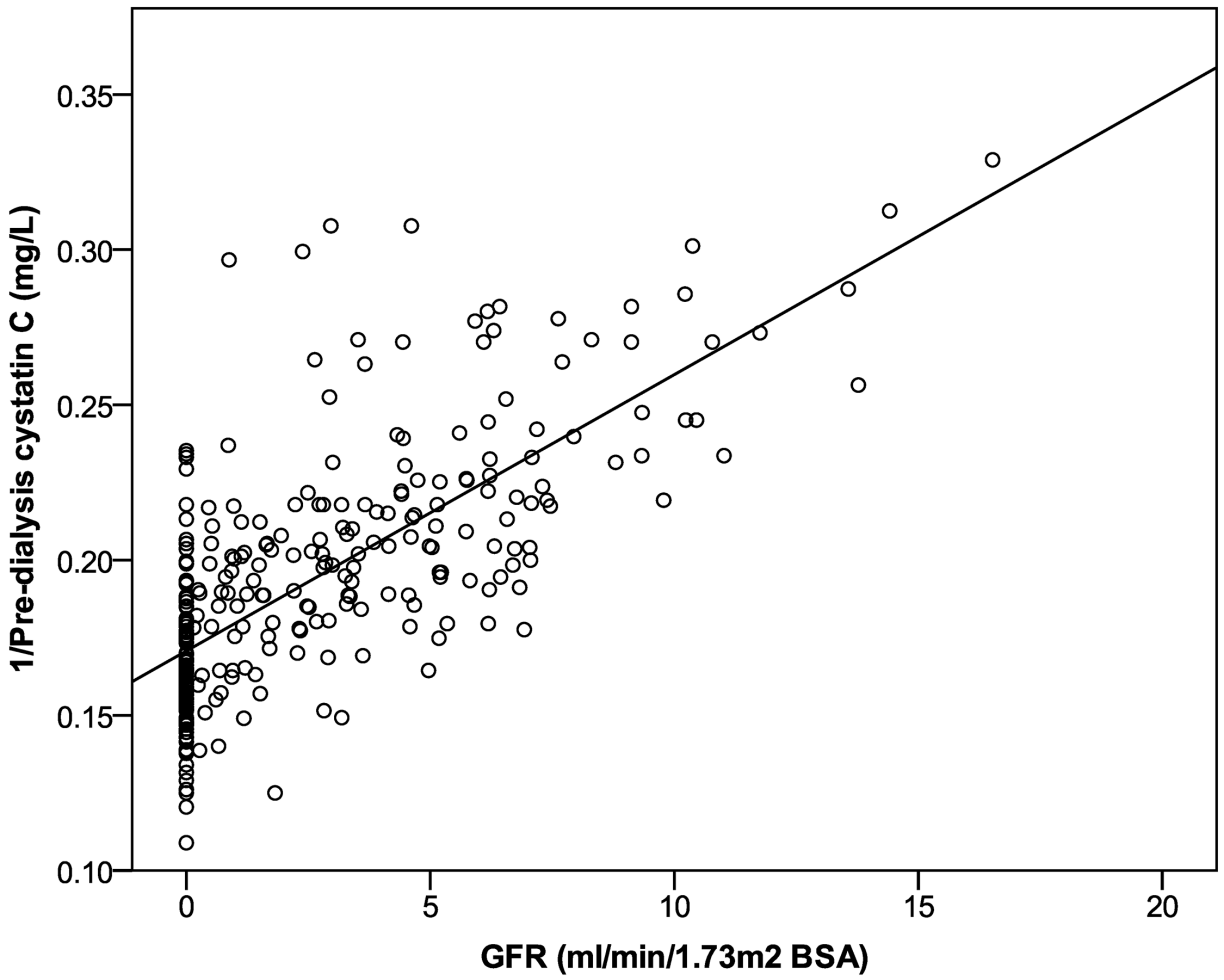
Relationship of GFR with the reciprocal of pre-dialysis plasma Cystatin C levels. GFR shown is calculated from the mean of urea and creatinine clearance. For the linear regression shown r^2^ was 0.5.

The parameter correlating best with GFR_Residual_, urea clearance and creatinine clearance was the reciprocal of pre-dialysis HD1 β2-microglobulin (r^2^ 0.67, 0.57, 0.68 respectively, Table 4). The reciprocal of pre-dialysis HD1 Cystatin C level correlated to a lesser degree (r^2^ 0.50, 0.44 and 0.48 for respectively). These relationships are shown in Figs 1 and 2. The correlations between the interdialytic rise (post HD1 to pre HD2) in both plasma β2 microglobulin and Cystatin C levels and GFR_Residual_, urea and creatinine clearances, were weaker (Table 4).

**Table 4:** Correlation of pre-dialysis Cystatin C and β2 microglobulin plasma levels and their interdialytic rise with various measures of RKF

		Cystatin C	β2 microglobulin	1/Cystatin C	1/ β2 microglobulin	Interdialytic rise in Cystatin C	Interdialytic rise in β2 microglobulin
	Pearson Correlation	-0.66	-0.72	0.71	0.82	-0.56	-0.69
GFR_Residual_ (ml/min/1.73m^2^)	r^2^	0.433	0.517	0.503	0.672	0.311	0.482
	Sig. (p)	<0.001	<0.001	<0.001	<0.001	<0.001	<0.001
	Pearson Correlation	-0.62	-0.69	0.66	0.76	-0.53	-0.67
Urea clearance (ml/min/1.73m^2^)	r^2^	0.39	0.47	0.44	0.57	0.28	0.44
	Sig. (p)	<0.001	<0.001	<0.001	<0.001	<0.001	<0.001
	Pearson Correlation	-0.62	-0.68	0.69	0.82	-0.53	-0.66
Creatinine clearance (ml/min/1.73m^2^)	r^2^	0.39	0.46	0.48	0.68	0.28	0.44
	Sig. (p)	<0.001	<0.001	<0.001	<0.001	<0.001	<0.001

GFR_Residual_ = the measured residual GFR (mean of urea and creatinine clearances).r^2^ is the square of the Pearson correlation coefficient.

Linear regression equations for GFR_Residual_ corrected to 1.73m^2^ based on the reciprocal of pre-dialysis β2 microglobulin and Cystatin C levels were computed as follows:

$${GFR}_{Residual}=slope\;\cdot \left(\frac{1}{PreHD1\;level}\right)+constant$$ (Equation 1)

where for β2 microglobulin (mg/L) slope = 160.3 (CI 147.5–173.1) and constant = -4.2 (CI -4.8 to -3.6), r^2^ = 0.67. For Cystatin C (mg/L) slope = 56.4 (CI 49.9–62.9) and constant = -8.4 (CI -9.6–-7.1), r^2^ = 0.50.

The above model for GFR_Residual_ corrected to 1.73m^2^ BSA could be improved marginally (r^2^ 0.68) by inclusion of both reciprocal pre-dialysis β2 microglobulin levels and pre-dialysis weight:

$${GFR}_{Residual}=A\cdot \left(\frac{1}{PreHD1\;\beta 2m}\right)+B\cdot \left(\frac{1}{PreHD1\;creatinine}\right)+C\cdot Weight+constant$$ (Equation 2)

where units are β2-microgloculin (mg/L), creatinine(μmol/L), weight(kg). In this model regression parameters were A = 142.2(CI 125.6 to 158.9), B = 899.8 (CI 309.3 to 1490.4), C = 0.013 (CI 0.002 to 0.024), constant = -5.63 (CI-6.70 to -4.55). Addition of other demographic variables such as age did not improve the model further.

Regression parameters were very similar if patients who were anuric were excluded. For instance, for Eq 1 based on reciprocal β2-microglobulin level the model r^2^ was 0.61 where we excluded these patients compared to 0.67 otherwise.

### Use of cut-off plasma levels of Cystatin C and β2-microglobulin to identify patients with significant residual kidney function

**Fig 3:**
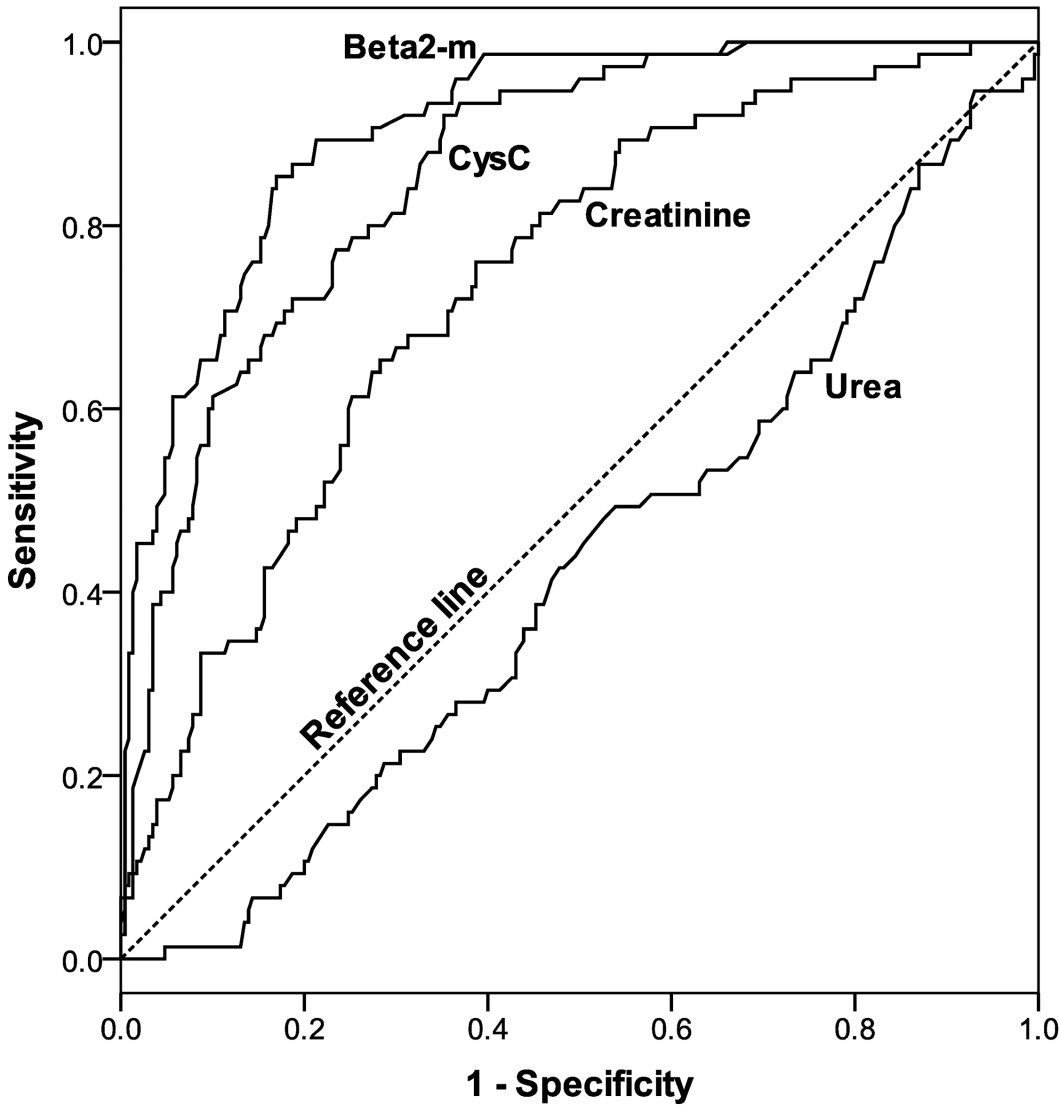
ROC analysis of pre-dialysis levels of Cystatin C, β2-microglobulin, urea and Cystatin C to identify patients with significant residual renal urea clearance ≥2ml/min/1.73m^2^ BSA. The greatest area under the curve was for β2-microglobulin.

ROC curves for pre-dialysis plasma Cystatin C, β2-microglobulin, urea and creatinine as indicators of residual kidney urea clearance ≥2ml/min/1.73m^2^ BSA are shown in **Fig 3**. Areas under each curve were 0.91 (CI 0.87–0.94) for β2-microglobulin, 0.86 (CI 0.82–0.90) for Cystatin C, 0.74 (CI 0.68–0.80) for creatinine and 0.43 (CI 0.35–0.50) for urea.

Using ROC analyses, for various levels of RKF considered significant (urea clearance 1-5ml/min and GFR 1-10ml/min/1.73m^2^ urea clearance), the cut-off level of β2-microglobulin was determined below which patients with each level of RKF could be identified with >90% specificity (false positive rates of <10%). Table 5 and Table 6 show for each relevant cut-off level the sensitivity and Area Under the Curve of the ROC analysis. It can be seen that identifying patients with urea clearance ≥4 ml/min/1.73m^2^ BSA using a β2 microglobulin level cut-off of ≤16.3 mg/L had 100% sensitivity and 90% specificity (Table 5). Likewise a cut-off of ≤16.7mg/L identified patients with a GFR ≥ 10 ml/min/1.73m^2^ BSA with similar levels of sensitivity and specificity (Table 6). However, for both clearances, identifying patients with lower levels of RKF had much lower sensitivity.

**Table 5:** Β2-microglobulin level cut-off levels that may be used to identify patients with residual renal urea clearance. For various levels of residual urea clearance, the cut-off β2-microglobulin level was determined that would yield a false positive rate (specificity) of 10%. For each level of urea clearance area under the curve (AUC) is shown for ROC analyses.

RKF level (ml/min/1.73m^2^ urea clearance)	Number of patients with RKF ≥ level in column 1 in cohort	Cut-off β2m microglobulin level (mg/L) for positive test	Sensitivity	Specificity	AUC
1	139	≤23.30	0.72	0.90	0.92
2	79	≤19.15	0.65	0.90	0.91
3	46	≤18.40	0.73	0.90	0.94
4	19	≤16.70	0.63	0.90	0.91
5	7	≤16.30	1.00	0.90	0.96

AUC: Area under the curve

**Table 6:** B2-microglobulin level cut-off levels that may be used to identify patients with residual renal function measured as GFR. For various levels of GFR the cut-off β2 microglobulin level was determined that would yield a false positive rate (specificity) of 10%. For each GFR level area under the curve is shown for ROC analyses.

(GFR in ml/min/1.73m^2^ BSA)	Number of patients with RKF ≥ level in column 1 in cohort	Cut-off β2m microglobulin level (mg/L) for positive test	Sensitivity	Specificity	AUC
1	155	≤24.05	0.7	0.90	0.89
2	130	≤23.65	0.76	0.90	0.92
3	104	≤21.80	0.77	0.90	0.91
4	84	≤20.40	0.75	0.90	0.92
5	64	≤19.05	0.67	0.90	0.91
6	48	≤18.85	0.71	0.90	0.93
7	29	≤18.05	0.83	0.90	0.95
8	18	≤17.45	0.94	0.90	0.96
9	16	≤17.35	0.94	0.90	0.97
10	11	≤16.70	1.00	0.90	0.98

AUC: Area Under the Curve

### Validation of methods to estimate RKF in the validation cohort

Mean age of the validation cohort was 59.6 years±SD17.2 and proportion of males 70% which did not differ significantly from the study cohort (p = 0.24 and p = 0.63).

**Fig 4:**
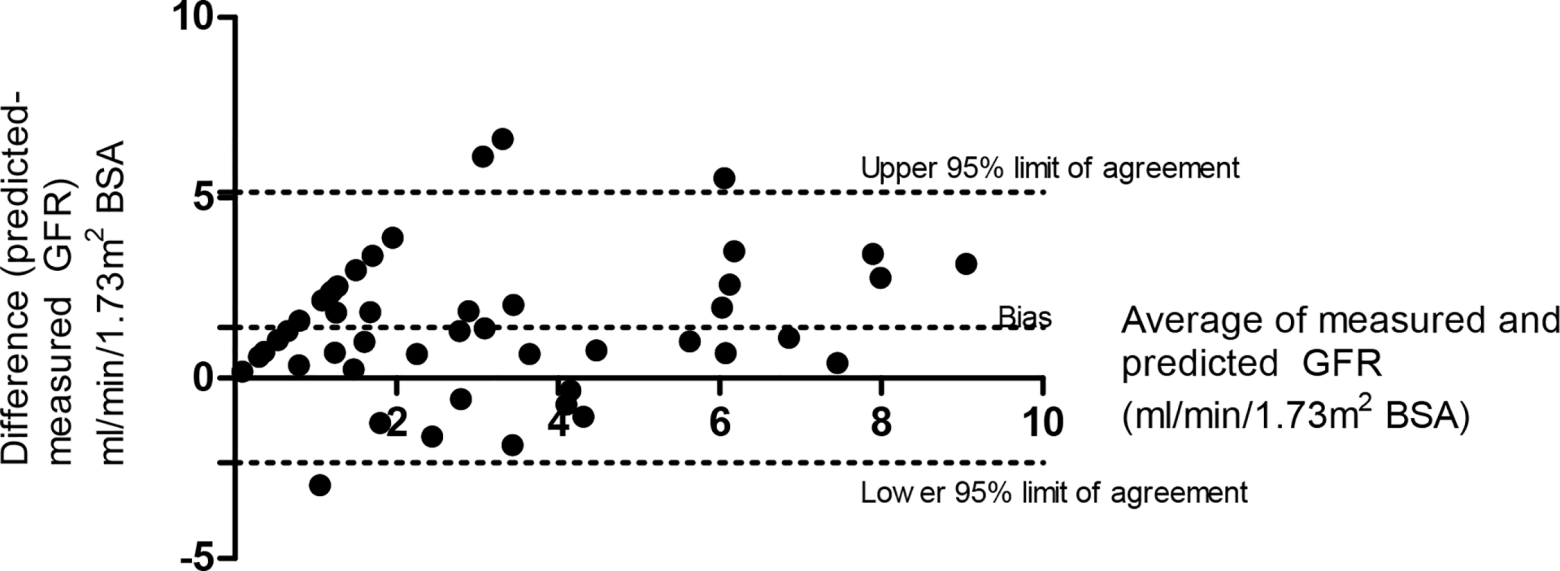
Bland-Altman analysis showing performance of Eq 1 for predicting GFR (ml/min/1.73m^2^ BSA) based on pre-dialysis β2-microglobulin plasma concentration.

The predictive model for GFR based on β2 microglobulin plasma level was applied to the validation cohort. The Pearson’s correlation r^2^ between predicted and measured GFR was 0.55. Using Bland-Altman analysis, the bias was 1.40 ml/min(±SD1.92) and 95% limits of agreement -2.35 to 5.16ml/min (**Fig 4**).

Use of the cutoff β2 microglobulin level of ≤19.15mg/ml (Table 5) to identify patients with urea clearance >2ml/min/1.73m^2^ BSA was tested in the validation cohort. Sensitivity was 54.2% and specificity 92.3%, similar to that predicted (Table 5). Positive predictive value was 86.7% and negative predictive value 68.6%.

## Discussion

In this study we have compared the potential use of two middle molecules to predict RKF. β2-microglobulin appears to be more closely correlated than Cystatin C with RKF. However, although reciprocal β2 microglobulin and Cystatin C in linear regression explain 67% and 50% of the variation in GFR respectively, their predictive value as plasma measures of RKF may be limited. This is because at lower levels of GFR, particularly <5ml/min, the relationship between their plasma levels and GFR_Residual_ is weaker. Inclusion of other parameters in the regression model (reciprocal creatinine concentration, weight) only marginally improved the regression model for GFR_Residual_.

Previously, Hoek *et al* determined a predictive equation for GFR in dialysis patients based on Cystatin C plasma concentration[6] but our data suggests that use of β2-microglobulin, or its reciprocal, holds more promise. We describe an algorithm to estimate GFR based on pre-HD β2-microglobulin: a simple regression using plasma concentration only. However, substantial variance remains unexplained and the performance of this equation in our validation cohort (r^2^ only 0.55) suggests that pre-dialysis plasma β2 microglobulin levels do not explain sufficient variance in GFR to provide an adequate clinical predictor on an individual patient basis. However, this equation may be useful as a research tool where an estimate of kidney function is required in a large cohort, for instance in survival analyses where correction for level of RKF is desired due to its large impact on outcomes.

However, the use of a cut-off pre-dialysis plasma level to identify patients with significant RKF shows more promise. KDOQI guidelines suggest permitting a reduction in minimum standard Kt/V for HD if urea clearance ≥2ml/min/1.73m^2^ BSA, and ignoring RKF below this level[25]. However, a lower level of ≥1ml/min/1.73m^2^ BSA has previously been associated with survival benefit [1]. Fig 3 demonstrates that pre-dialysis β2-microglobulin is most useful for identifying patients with significant RKF. A cut-off of <19.15mg/L is required to identify patients with ≥2ml/min/1.73m^2^ urea clearance while ensuring a low false positive rate of <10%, and we demonstrated its use in a validation cohort. This may allow for a reduction in minimum standard Kt/V target in selected individuals without the requirement for urine collection, though the sensitivity of this cut-off is low (65%).

It may be surprising that the interdialytic rise in Cystatin C was not more closely related to RKF, given that Cystatin C levels are less affected by muscle mass than creatinine and one might expect the magnitude of inter-dialytic rise to be predominantly defined by RKF. However at low GFR the non-renal Cystatin C clearance, estimated at 22.3ml/min/1.73m^2^ dominates over renal clearance in advanced kidney failure and is subject to wide inter-individual variation [24, 30]. it is not surprising therefore that a single pre-dialysis plasma concentration of this molecule is not the optimum marker for estimating GFR.

Use of reciprocal of β2-microglobulin or Cystatin C plasma levels in regression equations to predict RRF may be useful as research tools such as in prospective studies of survival in HD where correction for level of RRF is important. Serial plasma measurement of these molecules could, in survival analyses, allow for correction of RRF and take into account its time-dependent variation without the need to perform repeated urine collections for clearance.

A further potential use of β2-microglobulin or Cystatin C may be as markers of improving kidney function in subjects with acute kidney injury requiring dialysis. A plasma β2 microglobulin level of ≤16.7mg/L identified among the dialysis population those with GFR≥10ml/min/1.73m^2^ BSA with a low 10% false positive rate (Table 5). This potentially clinically useful cut-off level should be further explored and validated in the context of acute kidney injury. However, the numbers of patients with GFR≥10ml/min was low in the sample and we advise considerable caution with the sensitivities and specificity reported at this GFR cutoff.

In our study, we included all established patients on dialysis and did not exclude those who were anuric, or who had low level urine volume. In our view it was essential to include such patients to understand the limitations of using middle molecules to predict very low level RKF. Even low level RKF (urea clearance >1ml/min/1.73m^2^ BSA) appears to confer a large survival benefit and it is likely to be useful to identify such individuals[1]. Excluding such patients would have limited the applicability of our models in such patients with low level kidney function.

In this study all patients were treated with either HDF or high-flux HD. Convective volumes are much greater in HDF though in high-flux HD a degree of internal convection occurs[31]. We demonstrated that β2 microglobulin levels were lower in patients treated with HDF compared to high-flux HD in the subgroup whose GFR was <1ml/min/1.73m^2^ BSA which demonstrated again the dominant effect of RKF in defining plasma levels and perhaps explains why in the ESHOL study where a large proportion of patients would have had RKF no substantial difference in β2 microglobulin levels was found between high-flux HD and HDF groups[21]. Nevertheless, the substantial effect of HDF in increasing β2-microglublin clearance suggests that cut-off levels for this molecule used to identify significant GFR might differ in the high-flux HD and HDF populations, and this is confirmed by the contribution of total convective volume to the prediction of GFR in Eq 2. However addition of HDF to Eq 1 did not improve its predictive value.

This study has a number of limitations. The large number of patients precluded measurement of GFR with a “gold-standard” measure such as 51-Cr-EDTA. Although measuring GFR from interdialytic urine collections has limitations, these were reliably performed in our unit where, as part of our incremental HD programme, patients are well-accustomed to performing monthly urine collections. There is a paucity of data available on week-to-week GFR variation on HD, but this study did not investigate this aspect, instead aiming to determine whether a single plasma sample predicts GFR at a specific time point. Our study had did not have an external validation cohort and our regression algorithms require validation in other dialysis populations. Our regression models for RKF based on pre-dialysis β2 microglobulin concentrations were derived in our population dialysing with high-flux membranes and models are not generalizable to patients using low-flux membranes in whom plasma concentrations may be higher. However, as RKF is the dominant predictor of plasma β2 microglobulin concentration we suggest that even in the low flux setting it may be a useful (or even better!) marker of RKF. An additional limitation, specific to Cystatin C, is that this molecule forms dimers as part of the normal cellular processing pathway [32]. The degree to which these are measured by Cystatin C assays including the one employed in this study is unknown. This is important since Cystatin C dimerisation and binding to proteins such as C4 complement[33] may reduce the effective Cystatin C available for removal during dialysis, and is likely to enhance the post-dialysis rebound for this molecule. The methodology used to measure Cystatin C in our study was particle-enhanced DAKO immunoturbidimetric immunoassay whereas other techniques employed have included the immunonephelometric immunoassay[6]. Although these correlate highly (r = 0.97) bias exists[34] so the predictive models for GFR described by us may be specific to our measurement technique. The DAKO method has the advantage that the analytical coefficient of variation is lowest in the pre-dialysis cystatin C range observed in patients on HD[34].

With regard to β2-microglobulin, there are limitations to its use as a predictor of RKF which should be taken into account. CRP has been shown to related to its plasma level in multivariate analysis[16], is elevated in certain inflammatory conditions including HIV infection[35] and also in haematological conditions including myeloma[36]. It is subject to significant variation in non-renal clearance and is sequestered in body compartments which make its kinetics complex[37].Nonetheless, although its exhibits large post-dialysis rebound in both high flux HD and HDF, like Cystatin C it does appear to reach plateau in the inter-dialytic period so its levels reach equilibrium[24] and at this point plasma concentration is likely to reflect the balance of production and clearance. The relatively large inter-individual variation in plasma concentration in patients whose RKF is zero seen with both β2 microglobulin(Fig 1) and cystatin C is likely to therefore represent individual variation in generation rate and non-renal clearance which limits their use as a markers of RKF at the very low range GFR. However, changes over time in plasma concentrations of β2 microglobulin may more closely reflect RKF in individual patients and this requires further exploration.

Although the purpose of our study was to explore use of middle molecules to predict RRF in the dialysis population, a further proposed use might be as markers of dialysis clearance[38]. Accumulation of β2-microglobulin is harmful in end-stage renal failure with dialysis-related amyloid [39, 40]. Whether Cystatin C accumulation is harmful is currently a topic of interest, with recent data showing an association between Cystatin C level and cardiovascular mortality [41].The extent to which plasma concentration of these markers is affected by inflammation is unclear, but it is known that β2-microglobulin concentration is related to CRP levels[16].

In conclusion, although there has been considerable interest on use of Cystatin C for predicting GFR, our study suggests that in the dialysis setting β2-microglobulin plasma concentration is a superior marker. The limitations of using Cystatin C for predicting GFR in advanced CKD and the dialysis population relate to unpredictable non-renal clearance[24]. Although we report regression models for GFR based on pre-dialysis concentrations of these middle molecules, we advise significant caution if these are used on an individual patient basis particularly due to accuracy at low levels of RKF. However, cut-off levels of β2-microglobulin pre-dialysis plasma concentration may be more useful clinically to identify those with significant kidney function (urea clearance≥2ml/min/1.73m^2^ BSA) for the purpose of incremental haemodialysis, recognising that sensitivity of this test is limited. For identifying a higher level of kidney function (GFR≥10ml/min.1.73m^2^ BSA), that might indicate potential independence from dialysis, sensitivity and specificity are higher and use of pre-dialysis β2-microglobulin concentration for this purpose should be further explored.

## Supporting Information

S1 Dataset Raw data file. Raw data is shown for the modelling and validation cohort in Excel format.
(ZIP)
